# In vivo targeting of HIV gag to dendritic cells in combination with poly ICLC is safe and immunogenic in healthy volunteers

**DOI:** 10.1186/1742-4690-9-S2-O51

**Published:** 2012-09-13

**Authors:** M Caskey, C Trumpfheller, S Pollak, L Sinnenberg, A Hurley, J Pring, I Shimeliovich, B Yipp, N Anandasabapathy, S Mehandru, P Sarma, R Koup, R Bailer, G Tomaras, A Sato, T Keler, R Steinman, S Schlesinger

**Affiliations:** 1 The Rockefeller University, New York, NY, USA; 2 Duke University, Durham, NC, USA; 3 Statistical Center for HIV/AIDS Research & Prevention (SCHARP), Seattle, WA, USA; 4 Celldex Therapeutics, NJ, USA

## Background

In vivo delivery of HIV antigens within α-DEC 205 antibodies to maturing dendritic cells (DCs) in combination with maturation stimuli is a potential new vaccine platform. This phase-I study evaluates the safety and immunogenicity of DEC-targeting of HIV gag p24 in combination with poly ICLC in healthy volunteers.

## Methods

45 volunteers aged 18-60 were enrolled. 9 volunteers per dosage group (low: 0.3mg; mid: 1.0mg; high: 3.0mg) received α-DEC205-HIVp24 mAb plus a fixed dose of poly ICLC s.c., 3 volunteers received poly ICLC only, and 3 volunteers received saline, in a randomized double-blinded dose escalation design. Volunteers were vaccinated at weeks 0, 4, 12 and followed for 12 months.

## Results

Study remains blinded. Transient local and systemic reactogenicity occurred, without vaccine-related serious adverse events to date. Gag p24-specific IgG was induced in 9/15 (60%, 9 received vaccine plus adjuvant) volunteers in both low dose and mid dose groups at weeks 4, 8, 12, and 16. IgG titers were higher in the mid dose group and responses persisted for at least 6 months after last low dose immunization. Gag-specific CD4+ T cells were also detected following immunizations. IL-2 and TNF-α were the predominant cytokines. For CD4+ cells producing IL-2 or TNF-α, the response rates ranged from 33 to 46% (5-7/15 volunteers, 9 received vaccine plus adjuvant) and 8 to 40% (1-6/15 volunteers) post-vaccination in the low and mid dose groups respectively. Among positive responders, the median magnitude across visits ranged from 0.09% to 0.23% in the low dose group and from 0.06% to 0.17% in the mid dose group.

## Conclusion

This novel DC-targeted protein HIV vaccine in combination with poly ICLC is safe and immunogenic in humans. Cellular and humoral immune responses are induced. Antibody responses are durable, with antibody titers unchanged at 6 months following last immunization.

